# Increased Caspase-3 Immunoreactivity of Erythrocytes in STZ Diabetic Rats

**DOI:** 10.1155/2012/316384

**Published:** 2012-04-26

**Authors:** Uğur Fırat, Savaş Kaya, Abdullah Çim, Hüseyin Büyükbayram, Osman Gökalp, Mehmet Sinan Dal, Mehmet Numan Tamer

**Affiliations:** ^1^Department of Pathology, School of Medicine, Dicle University, Diyarbakır 21000, Turkey; ^2^Department of Immunology, School of Medicine, Dicle University, Diyarbakır 21000, Turkey; ^3^Department of Hepatology and Transplantation, Division of Gene and Cell Based Therapy, School of Medicine, King's College London, London SE5 9NU, UK; ^4^Department of Medical Pharmacology, School of Medicine, Dicle University, Diyarbakır 21000, Turkey; ^5^Department of Internal Medicine, Division of Endocrinology, School of Medicine, Dicle University, Diyarbakır 21000, Turkey; ^6^Department of Internal Medicine, Division of Endocrinology, School of Medicine, Süleyman Demirel University, Isparta 32260, Turkey

## Abstract

Eryptosis is a term to define apoptosis of erythrocytes. Oxidative stress and hyperglycemia, both of which exist in the diabetic intravascular environment, can trigger eryptosis of erythrocytes. In this experimental study, it is presented that the majority of erythrocytes shows caspase-3 immunoreactivity in streptozocin- (STZ)-induced diabetic rats. Besides that, caspase-3 positive erythrocytes are aggregated and attached to vascular endothelium. In conclusion, these results may start a debate that eryptosis could have a role in the diabetic complications.

## 1. Introduction

Hyperglycemia and oxidative stress are the prominent features of diabetes mellitus (DM) and seems to play a crucial role in DM-related microvascular complications. In addition, complications of DM like nephropathy, retinopathy, and macrovascular disease are associated with anemia [1, 2].

Eryptosis, a term used for apoptosis of erythrocyte, is triggered with osmotic shock, oxidative stress, or energy depletion [3]. Moreover, eryptosis is characterized with cell shrinkage, membrane blebbing, membrane phospholipids scrambling, and phosphatidylserine (PS) shifting from inner to outer membrane of the erythrocyte [4]. It is demonstrated that death receptor initiated pathway of apoptosis takes a role in eryptosis involving Fas, caspase-8, and caspase-3 [5]. Caspase-3, an executioner caspase, immunoreactivity is observed in the lysate of erythrocytes obtained from type 2 DM patients [6]. Besides that, previous reports show the evidence that eryptosis underlies anemia and microvascular injury both of which may be related with endothelial adhesion and increased aggregation of erythrocytes, in DM patients [3, 7–9].

In this study, it is presented that increased caspase-3 activity is detected in erythrocytes in the vasculature of cerebrum and cerebellum of STZ-induced DM rats. In other words, eryptotic erythrocytes number is increased in DM rats. This finding can explain the anemia and the underlying or accompanying factors of microvascular injury, such as erythrocyte aggregation and endothelial erythrocyte adhesion in DM.

## 2. Materials and Methods

### 2.1. Animals

Female Wistar Albino rats are obtained from the Laboratory Animals Facility of Dicle University. In this study, rats are handled in accordance with the Animal Welfare Act and the Guide for the Care and Use of Laboratory animals prepared by the Animal Ethical Committee of Dicle University. Rats are distributed into following groups with *n* = 7 each: non-DM group and DM group. Rats in non-DM group received citrate buffer intraperitoneal (i.p.) injections. Rats of DM group were injected with STZ (50 mg/kg, i.p.; in 0.1 M citrate buffer, pH 4.5) for induction of DM. Blood glucose level of rats in DM group is confirmed before sacrifice and it is over 250 mg/mL. Thirty days after i.p. administration rats are executed for the analysis.

### 2.2. Biochemical Analysis

The excised cerebrum for biochemical analyses were weighed, immediately stored at −80°C. The cerebral tissues are perfused with 1.15% ice-cold KCl (w/v) and sliced into minute pieces then homogenized in five volumes of the same solution. The homogenate is centrifuged at 14.000 rpm at 4°C for 30 minutes (min). The supernatants are used for the assay. Lipid peroxidation level, indicator of oxidative tissue damage, in the cerebrum is defined with malondialdehyde (MDA) amount as mentioned by Ohkawa et al. [10].

### 2.3. Immunohistochemical Staining

Cerebrum and cerebellum are fixated in 10% formaldehyde for 48 hours. Then, they are dehydrated and embedded in paraffin. Paraffin blocks are sliced in 4 *μ*m thickness with microtome. Tissue slices are located on positive-charged glasses and incubated at 60°C for 60 min to deparaffinize. Then, slides are treated with xylene (3 × 5 min) and hydrated with alcohol. Antigen retrieving process is done in citrate buffer (10 mM Citric Acid, 0.05% Tween 20, pH 6.0) by boiling and cooling down (x3) in microwave oven. After this and every other processes slides are washed with Phosphate-Buffered Saline twice for 5 min each. Endogenous peroxidase blocking is done with Peroxide Block (ACA500, ScyTek, UT, USA) for 10 min at room temperature (RT). Then, slides are treated with Super Block (AAA125, ScyTek, UT, USA) for 20 min at RT. Then, slides are treated with rabbit anti-human Caspase-3 polyclonal Antibody (1 : 750) (cat#. GTX73090, Gene Tex, Inc.; CA92606, USA) cross reacting with rat caspase-3 for 20 min at RT. Then, biotinylated SensiTek polyvalent antibodies (ABF125, ScyTek, UT, USA) are applied for 20 min at RT before SensiTek HRP, streptavidin-HRP complex (ABG125, ScyTek, UT, USA) treated for 20 min at RT. Finally, AEC Chromogen/Substrate Bulk Kit (ACJ125, ScyTek, Utah, USA) working solution is applied for 10 min at RT. Counterstaining is performed with Mayer hematoxylin (cat#. 05-M06002, Bio-Optica, Milano, Italy). Slides are evaluated under the light microscope (Nikon ECLIPSE 80i, Japan) at ×400 magnification by a pathologist blinded to study groups.

### 2.4. Statistics

Caspase-3 positive and negative erythrocytes are counted in vascular spaces at randomly selected 8 regions for each slide. The averages of percentage of positive cell are calculated for each slide. Then, the means of percentages of positive cells of the groups are compared with Student's *t*-test. The difference of the means of MDA levels is also calculated with Student's *t*-test. Data are presented as mean ± S.D.

## 3. Results

**Figure 1:**
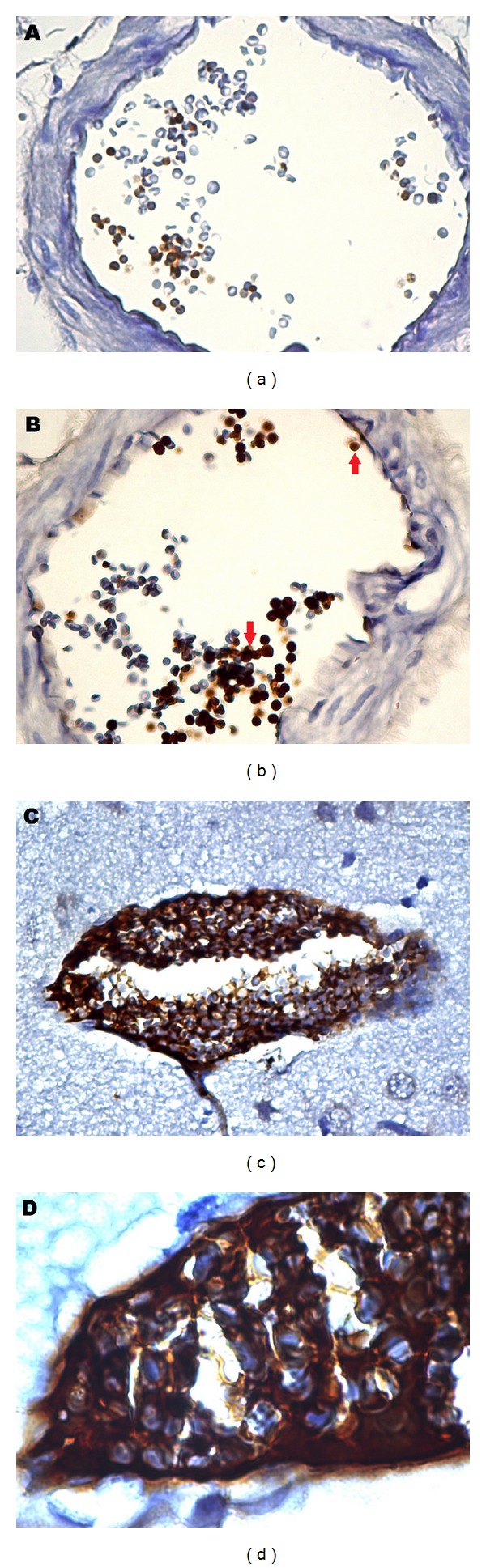
Caspase-3 immunoreactivity of erythrocytes (immunoperoxidase). Baseline caspase-3 positivity of erythrocytes in rats of non-DM group (a). Red arrows show caspase-3 positive erythrocytes in brown color in rats of DM group (b). Caspase-3 positive erythrocytes, aggregated and adhered to vascular endothelium in diabetic rat (c). Caspase-3 positive erythrocytes occluding vascular spaces presented in higher magnification endothelium in diabetic rat (d). Magnifications are 400 in (a), (b), and (c), and 1000 in (d).

**Figure 2:**
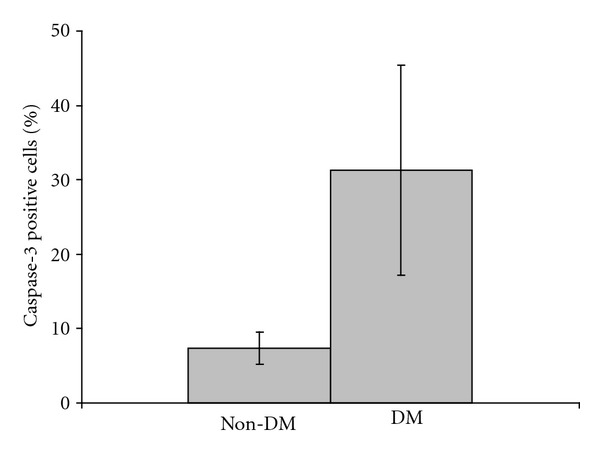
Percentages of Caspase-3 immunoreactive erythrocytes in diabetic and nondiabetic rats. Means ± SD of the percentages of caspase-3 positive cells are compared with student's *t*-test (*P* < 0.0001; *n* = 7).

In this study, MDA levels, showing lipid peroxidation representing oxidative tissue destruction, in cerebral tissues are significantly higher in DM group than non-DM group, 451 ± 66 nmol/gr protein, and 263 ± 55 nmol/gr protein, respectively (*P* < 0.0001). In addition, immunohistochemical staining of the cerebral and cerebellar tissues demonstrates that a few number of erythrocytes show immunoreactivity to caspase-3 in non-DM group (Figure 1(a)), that is physiological outcome of senescence of erythrocytes, possibly. However, the number of capase-3 immunoreactive erythrocytes is elevated in DM group (Figure 1(b)). In addition, majority of the erythrocytes with caspase-3 immunoreactivity attached each other in DM group (Figure 1(c)). Furthermore, these aggregated erythrocytes adhered to endothelium of the vessels (Figure 1(c)). What is more, some of the vessels are totally occluded with caspase-3 positive erythrocytes in these rats in DM group (Figure 1(d)). The statistical picture of our finding is as follows: 31.33 ± 9.03% of the erythrocytes show immunoreactivity to caspase-3 in DM group; nonetheless, 7.43 ± 3.36% of the erythrocytes stained with caspase-3 in non-DM group (Figure 2). In addition, the mean of percentages of caspase-3 positive cells is significantly different in DM group than other group (*P* < 0.0001). These findings suggest that eryptosis, ignited with either high serum glucose level or oxidative stress or bought of them and defined with prominent caspase-3 immunoreactivity, is a considerable underlying cause of the diabetic complications, such as microangiopathy and anemia.

## 4. Discussion

In this study, in brief, caspase-3 immunoreactivity in erythrocytes, aggregation, and endothelial adhesion of erythrocytes are shown with immunohistochemical staining of cerebral and cerebellar tissues in the diabetic rats. In diabetic rats, presence of caspase-3 immunoreactivity in erythrocytes may be an indirect evidence of eryptosis accompanying conditions like PS exposure and caspase-8 activity.

Up to date, according to our literature search, this is the first report that demonstrates caspase-3 activity in erythrocytes with immunohistochemical study in diabetic rat. Beside that, there are two other reports supporting our finding, caspase-3 activity in erythrocytes [6, 11]. First report presents that caspase-3 activity is significantly higher in type 2 DM than healthy subjects [6]. Second report shows that even erytrhocytes of type 2 DM patients without chronic kidney disease are stained with annexin V, bind PS, and show early apoptotic cells [11]. Our results suggest that hyperglycemia, a kind of hyperosmalar state, and oxidative stress may initiate the cascade of eryptosis. Hyperglycemia and oxidative stress are a well-documented trigger of eryptosis; however, how they do succeed that is not clearly demonstrated yet [6]. One explanation of that may be death receptor based. Previously, it was reported that Fas, caspase-8, and caspase-3 exist in erythrocytes and take role in eryptosis [5]. In the same study, it is also reported that erythrocytes expresses FasL. In the line with this and our result, it may be thought that hyperglycemia and oxidative stress direct erythrocytes into hemolytic pathway, very parallel to eryptotic pathway. Then, PS is exposed on the outer membrane of the erythrocyte during hemolysis which results in erythrocyte-to-erythrocyte attachment. Thus, Fas-FasL interaction starts eryptosis, which may protect microcirculation from occlusion and be pointed with caspase-3 positivity, in erythrocytes to escape hemolysis in the process of erythrocyte aggregation. In short, eryptosis may work as a mechanism saving erythrocytes from hemolysis. On the other hand, hyperglycemia and oxidative stress may induce eryptosis independent of hemolysis beginning with death receptor pathway.

It is clearly seen in our study that diabetic rat erythrocytes attached each other and endothelial surface (Figure 1(c)). In reports, increased aggregability was observed in the red blood cells of diabetic patients [8, 9]. In one report, it is shown that PS decreases energy to need erythrocyte-erythrocyte attachment [7]. In addition to these reports, it is claimed that PS exposure is responsible for increased erythrocyte adhesion to endothelium in central retinal vein occlusion [12]. Here, we do not present direct evidence of PS presence on erythrocytes; nonetheless, caspase-3 immunoreactivity may be accepted as indirect evidence of its existence. As a result, we conclude the reason of increased aggregability and adhesiveness may be PS presence in outer membrane of eryptotic erythrocytes. Besides all, it is worthy to say that caspase-3 immunoreactivity is observed in the vascular endothelium of the cerebrum and cerebellum in many areas (unpresented data) in DM group. In line with this, high aggregability and adhesiveness of erythrocytes may cause vascular occlusion which may explain the underlying pathology in microangiopathic complications of diabetes.

Anemia in diabetics is generally overlooked and thought to be developed due to nephropathic complication of diabetes. In addition, low or nonfunctional erythropoietin is accused of anemia [1, 13]. On the other hand, hyperglycemia itself can be the reason of anemia if diabetic treatment is not given properly or absent. As an example, we have seen once a case: 70-year-old female who is diagnosed as having diabetes without nephropathy after hospital admittance is presented with severe anemia (hemoglobin = 8 g/dL) and recovered from anemia (hemoglobin = 12.5 g/dL) with diabetic prescription (unpublished case). We believe that our result, high frequency of eryptotic erythrocytes in diabetics, is a reflection of functional anemia, undetectable with routine laboratory tests, which is also mentioned elsewhere as anemia masked by dehydration [14]. Consequently, to diagnose anemia in diabetics, calculation of intravascular total volume with red blood cell count may be taken into consideration. Alternatively, caspase-3 positive erythrocyte count may be another solution to diagnose functional anemia in diabetics.

## 5. Conclusions

In short, it is presented here that the number of eryptotic erythrocyte in diabetic rat is higher than non-DM group. This result may help us to understand the bases of anemia and microangiopathy in diabetics. In conclusion, the treatment of masked anemia in diabetes may lead to improvement of diabetic complications in these patients.

##  Conflict of Interests

All authors fully disclose that there is no financial or ethical conflict of interest.
